# Time for Action: Verbal Action Cues Influence Temporal Binding

**DOI:** 10.3389/fpsyg.2020.00160

**Published:** 2020-03-04

**Authors:** Tom G. E. Damen, Rick B. van Baaren, Ap Dijksterhuis

**Affiliations:** ^1^Department of Psychology, Utrecht University, Utrecht, Netherlands; ^2^Department of Social Psychology, Behavioural Science Institute, Radboud University Nijmegen, Nijmegen, Netherlands

**Keywords:** control perception, priming action, social cueing, temporal binding, agency

## Abstract

Prior research has shown that our perception of time is compressed when we volitionally perform actions, a phenomenon referred to as temporal binding. In three studies, we investigated the degree to which contextual cues that signaled other agents and related to actions would influence binding, given that those cues may affect individual’s feelings of independent action performance. Participants heard action verbalizations that did or did not match actions that participants had already begun performing. Participants’ time estimates of the intervals between action initiations and action effects were higher on trials in which they heard verbalizations that matched their ongoing actions, and lower on trials in which the verbalizations and actions did not match. Such effects did not occur when participants passively observed actions and effects being caused by the computer. These results show that the compatibility of action cues with ongoing actions influences temporal binding effects, suggesting that they influence our feelings of having been an independent agent.

## Introduction

Our perception of time is a highly subjective experience. Indeed, a long tradition in research has shown that the perception of time can be influenced by many different psychological factors (Benford, 1944; Hirsh and Sherrick, 1961; Libet, 1985; Angrilli et al., 1997). For example, Haggard et al. (2002) showed that when we act voluntarily, actions are perceived to occur later in time and action effects are perceived to occur earlier in time, making the time intervals between actions and action effects appear shorter for voluntary compared to involuntary actions. This phenomenon is referred to as intentional binding, or temporal binding, and has been repeatedly demonstrated in previous research (e.g., Engbert et al., 2007; Wenke and Haggard, 2009; Moore et al., 2009a,b; Ebert and Wegner, 2010; Obhi and Hall, 2011; Kühn et al., 2013).

Given the differences in temporal binding effects when contrasting voluntary and involuntary actions, temporal binding has become strongly linked to the sensation of volitionally performing actions and causing effects known as agency (Frith, 2013). This link is what makes temporal binding more than just an interesting phenomenon in fundamental research: As an implicit marker of volition, it is strongly related to cognitions about agency, responsibility, and ultimately, moral judgment. Temporal perception therefore covaries with – and can sometimes even inform – moral judgment (Moretto et al., 2011; Christensen et al., 2019). For example, we consider ourselves more responsible for outcomes that are perceived in close temporal proximity to our actions (e.g., Wegner, 2002). In similar vein, we typically perceive actions and outcomes to occur closer together in time when we also consider ourselves responsible (e.g., Desantis et al., 2011).

Binding occurs when agency is high, and actions and effects are perceived as volitionally produced (e.g., Desantis et al., 2011). In contrast, binding has been shown to be reduced or absent when actions and effects are externally produced (e.g., Haggard et al., 2002). In daily life there are, however, many situations in which there is not such a stark contrast between self- and externally produced actions. Although we *ourselves* may perform actions, we often do so in the context of others who at times subtly, and at other times very overtly, influence the way we act and the way we think about acting. Only a limited number of studies have however looked at the influence of social cues on temporal binding (e.g., Poonian and Cunnington, 2013; Inoue et al., 2017) and there are virtually no studies investigating binding in the context of social action cues (Caspar et al., 2016). In the present research, we therefore investigated whether the compatibility of social cues with ongoing actions would influence the perception of time for those self-produced actions.

### Premotor and Inferential Accounts of Binding and Agency

Binding occurs when participants willfully act (e.g., Engbert et al., 2008), and does not occur in situations when movements are externally induced (Haggard et al., 2002; Wohlschläger et al., 2003a; see also Engbert et al., 2007, 2008). Although the precise mechanism causing intentional binding is still a matter of empirical debate (e.g., Waszak et al., 2012; Hughes et al., 2013), traditionally both temporal binding and agency have been argued to emerge by dedicated motor control mechanisms predicting the sensory outcomes of actions (e.g., Blakemore et al., 1998, 2000; Blakemore et al., 1999; Wolpert and Ghahramani, 2000; Haggard et al., 2002; Engbert and Wohlschläger, 2007). To a large degree, binding is believed to be caused by joint simulations of motor actions and their predicted outcomes, causing temporal perception of the action to be moved toward the effect, and temporal perception of the effect moved toward the action.

Other approaches suggest binding and agency also emerge through inferential mechanisms (Wegner and Wheatley, 1999; Wegner, 2002, 2003; Moore and Haggard, 2008). Accordingly, binding and agency are influenced when we can easily relate our actions or the action effects we produce to any thoughts preceding it (David et al., 2011), and when our agency beliefs (Desantis et al., 2011) or the contextual information (Dijksterhuis et al., 2008) make us appear as the most likely agent.

The optimal cue integration approach (Moore and Fletcher, 2012) and other theoretical approaches (e.g., Synofzik et al., 2013) consider the premotor and inferential accounts as complementary. Both premotor and inferential cues are considered to be continuously integrated and weighted depending on their availability and reliability in a given situation – thereby influencing binding and agency.

### Inferential Manipulations of Temporal Binding

A number of studies have shown that temporal binding can be influenced by inferential manipulations. For example, Barlas and Obhi (2013) and Barlas et al. (2017) showed that a high number of action possibilities increased temporal binding compared to a low number of action possibilities. And Desantis et al. (2011) revealed that when participants had high beliefs about personal agency, they showed increased binding – of the effects toward the actions – compared to when participants believed their actions were caused by someone else (see also Lynn et al., 2014). These findings suggest that binding is influenced by manipulations that are (on face-value) not directly related to premotor prediction.

Manipulations that occur before action initiation – such as the manipulation described above – may nevertheless indirectly influence premotor prediction. For example, Rigoni et al. (2011) showed a relation between control-beliefs and premotor neural activity: When individuals were led to believe they generally had no personal control over events in their lives these individuals also showed reduced readiness potentials prior to action performance. The research by Rigoni et al. (2011) is a strong example of the way in which high-level manipulations (e.g., beliefs) may have low-level consequences (e.g., premotor activity). One further implication of this research is that it suggests that manipulations that are considered inferential in nature can nevertheless influence premotor prediction – thereby casting doubt on whether inferential mechanisms directly influence binding.

A way in which one can more convincingly test the unique contribution of inferential mechanisms is by introducing manipulations when actions have already been initiated. For example, a study by Moore and Haggard (2008) showed that binding could occur retrospectively on trials in which no effect was predicted but an effect occurred nevertheless. Moore and Haggard suggest that the emergence of an unexpected effect triggers *post hoc* inferential sense-making ultimately leading to a binding effect. However, whether binding is also influenced by inherently inferential manipulations (e.g., cues suggesting other agents) that are introduced after action-initiation is still unexplored.

Another possible way to establish the role of inferential processes in binding would be to investigate binding in situations in which motor predictions are less accurate, thereby increasing one’s reliance on inferential information (Moore and Fletcher, 2012). As we argue in the following section, situations of continuous action performance may provide exactly such a context.

### Continuous Action Performance

People are often required to perform ongoing actions for which they receive continuous feedback. For example, steering and pedaling your bike on the way to work can be perceived as one long action in which you are provided with a strong and rich flow of sensory information. However, the methodology of studies in the agency domain typically does not feature actions that take relatively long to resolve – instead actions and action outcomes are quick and discrete. As was argued by Wen et al. (2015), it is important that agency paradigms include longer actions as the processes underlying agency for discrete versus prolonged actions may differ. Specifically, from a motor prediction perspective it may be difficult to generate accurate predictions about sensory feedback for ongoing actions. Keeping to the previous example, biking to work would involve rich and continuous feedback, and due to the increased sensory input and overall complexity in this situation one’s predictions may become less specific – especially compared to discrete one-action one-outcome settings. In line with the optimal cue integration approach (Moore and Fletcher, 2012), such a reduced reliability from motor predictions can increase the degree to which people rely on inferential information in their judgment of agency. However, more studies that feature inferential manipulations and relatively longer actions are required to validate the notion that inferential information is important in such settings.

### Action Cues Influence Agency

In the agency domain a number of studies have investigated the degree to which cues related to actions can influence agency. Wenke et al. (2010) subliminally cued participants with arrow symbols pointing to the left or right just before participants were about to press a left or right button. Their results showed that when the direction of the arrows pointed toward the spatial location of the action, individuals reported increased agency compared to when the arrows did not point toward the action location. According to Wenke et al. (2010), the subliminally presented compatible primes enhanced the ability to select the appropriate action, and this action fluency then increased the sense of agency. A recent paper by Sidarus and Haggard (2016) reported similar results. Specifically, they showed that supraliminally presented arrow symbols pointing toward the compatible response locations in a Flanker task were related to increases in explicit agency ratings compared to arrow symbols pointing toward incompatible response locations. In conclusion, previous literature shows that cues that affect the fluency of action selection can influence the sense of agency. The question then remains whether action cues also influence measures of binding.

### Social Processes in Binding and Agency

Agency is influenced by the principle of exclusivity, which holds that it is important to our sense of agency to be able to perceive ourselves – and not others – as the most likely cause for actions and effects. Many of our actions are however performed in a social context: We imitate (Miller and Dollard, 1941; Wegner and Sparrow, 2004) and conform to others (Asch, 1956); we can be susceptible to suggestions from others (Bearden et al., 1989); and sometimes we are subject to direct commands of others telling us what to do (Kelley, 1972; Milgram, 1974). Social cues can therefore “threaten” our principle of exclusivity in ways that do not apply to non-social cues: While seeing an arrow pointing left may help us to press a left button and increase agency (Wenke et al., 2010; Sidarus and Haggard, 2016), an agent specifically telling us to go left may “threaten” the principle of exclusivity, and lead to a reduced sense of agency.

The unique influence of social action cues was shown in a paper by Damen et al. (2014). Specifically, in their paradigm they introduced another agent (“the computer”), and presented participants with action instructions (the words “left” and “right”). When the instructions were subliminally presented the participants reported increased agency when there was a match between instructions and actions (similar to the findings by Wenke et al., 2010; Sidarus and Haggard, 2016). However, when the instructions were presented supraliminally and could be consciously perceived, the effects reversed: The visible and compatible instructions lowered the sense of agency instead. Damen et al. (2014) argued that in all likelihood, the conscious awareness of the other agent and the presented instructions could have threatened the participants’ sense of having been an independent agent, resulting in a reduced sense of agency when their actions matched the instructions.

### Challenges

To reiterate, a number of important challenges remain to be addressed in the agency domain. First, although the principle of exclusivity is considered vital to the sense of agency (Wegner, 2002, 2003), only a small number of studies on agency in general – and on binding in particular – involve social cues. This reflects a gap in the literature that needs to be addressed, especially given the fact that a previous study measuring explicit agency suggests that social action cues decrease agency when the cues are consciously perceived and cued actions are subsequently performed (Damen et al., 2014). However, whether such compatibility effects emerge on implicit measures of agency (i.e., temporal binding) is unclear, thereby reflecting an important second challenge to address. Third, although inferential agency manipulations have been investigated using implicit measures (Desantis et al., 2011; Barlas et al., 2017), such manipulations are typically introduced before actions are performed. This makes it difficult to determine whether the processes underlying the reported effects are inferential in nature or actually emerge from motor prediction. Fourth, as agency methodologies typically involve short actions, there is a lack of knowledge on agency and binding when actions take long to resolve (Wen et al., 2015). The present research was designed to address these challenges.

### The Present Research

In the present research, we report three studies in which we aimed to investigate whether temporal binding for self-produced actions is influenced by the compatibility of action cues with those actions. To measure temporal binding, participants were required to act, and when they had concluded their action, they were asked to estimate and report the time it took from the moment they initiated their action until they caused an effect.

Participants were presented with verbal action cues. The verbalizations related to actions and not to action outcomes, making them less likely to influence processes of outcome prediction. Additionally, the verbalizations were presented after individuals had already initiated their actions, reducing the probability that predictive mechanisms play a role. Furthermore, verbalizations were introduced during continuous action performance, a context in which people may rely more on inferential cues (Wen et al., 2015).

To test whether action cues influence temporal binding, we manipulated the content of the verbalizations. In Study 1, participants heard verbalizations that were either compatible with the performed action or were in opposition to the performed action. The primary aim of Study 1 was to test the theory proposed by Damen et al. (2014), that acting “against” instructions or cues would be likely to increase the sense of having been an independent agent, whereas “following” suggestions is likely to threaten this view, and reduce agency instead. As such we expected that time interval estimates would be higher (indicating a lower sense of agency) when participants heard a verbalization that was compatible to the action they were performing, and time estimates would be lower (indicating a higher sense of agency) when opposite verbalizations were presented.

As we expected, these manipulations only to have effects in the context of action performance; in Study 2, participants were required to observe actions and action effects by another agent (the “computer”) while also being presented with compatible and opposite verbalizations. The primary aim of Study 2 was therefore to establish that compatibility effects only emerge in the context of action performance. As such, we expected no effects of the verbalizations considering that participants were not actively involved.

Finally, Study 3 was largely a replication of Study 1 besides that participants additionally heard verbalizations that were unrelated to the performed task. These verbalizations served as a control condition against which the directionality of (in)compatibility was investigated. Compatible primes may reduce agency, incompatible primes may increase agency, or both may apply. The primary aim of this study was to explore the direction of the compatibility effect.

## Methods

### Participants

Each study featured 50 undergraduate students who participated in exchange for a small fee (Study 1: 43 females; *M*_age_ = 22.28, age range 18–45; Study 2: 30 females; *M*_age_ = 22.34, age range 18–31; Study 3: 34 females; *M*_age_ = 22.44, age range 18–43). Although the majority of participants was female, gender is typically not a meaningful predictor in research on agency and binding. A participant involved in one study was excluded from participation in the other studies. The studies were approved by the Research Ethics Board of Utrecht University’s Faculty of Social Sciences and all participants provided written informed consent prior to beginning the study.

### Materials and Procedure

Participants were presented a red circle on their monitor, ostensibly representing a balloon. In Studies 1 and 3, participants were instructed to inflate the balloon until it popped. They could start inflating the balloon by clicking once on the red circle and holding down the mouse-button, thereby causing the red circle to increase in size. In Study 2, participants were not required to click and hold down the mouse-button. Instead of performing an action, participants saw the “computer’s cursor moving toward the balloon, then inflating the balloon until it popped.

After a fixed period of time (2.5 vs. 3.0 vs. 3.5 s), the balloon popped, indicated by the recorded sound of a balloon burst and the presentation of the picture of a popped red balloon for 0.5 s. Participants were told to release the mouse-button as quickly as possible when the balloon popped (In Study 1, we logged how quickly participants released the button after the balloon popped. This was after 0.314 s on average. There were no differences in release-times between the instruction conditions). Participants then used the mouse to give an estimation of the time it took for the balloon to pop from the moment they/the computer had started inflating it. Participants were required to provide their answer within a range of 2.0–4.0 s. In case participants did not hold down the mouse-button in Studies 1 and 3, and accidentally released it prior to the balloon burst, an error message appeared and the trial restarted.

**Figure 1:**
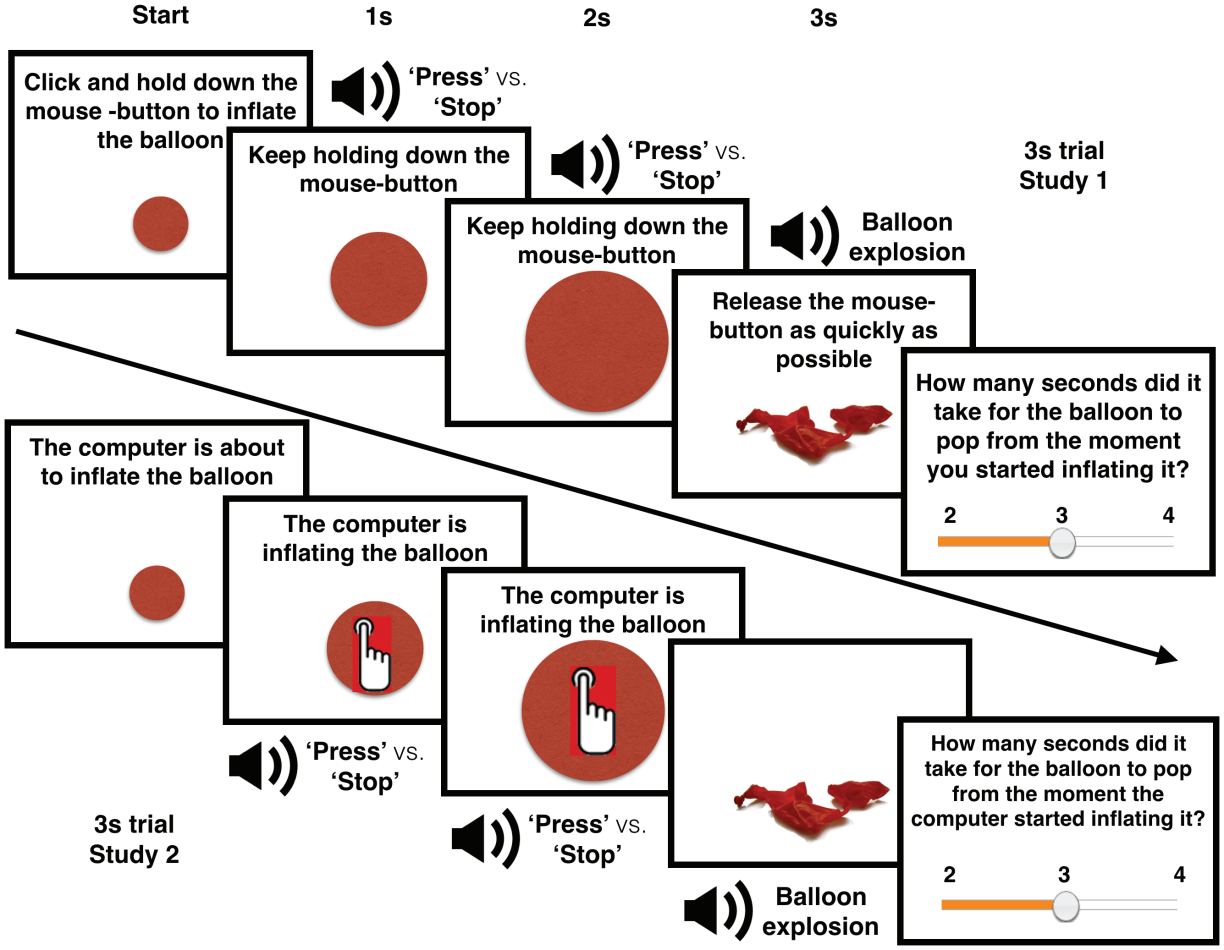
Experimental overview. The upper half depicts a 3.0 s trial from Study 1. The lower half depicts a 3.0 s trial from Study 2.

Participants were explained that during the inflation process, they would hear recorded voices delivered over a headset. Participants were informed that the voices were contextual and not task-relevant – they should listen to them, but not be influenced by them. Participants in Studies 1 and 2 would hear the words “Press” (compatible action verbalization) or “Stop” (opposite action verbalization) evenly divided over 60 trials. Participants in Study 3 would hear “Press” (compatible action verbalization); “Stop” (opposite action verbalization); or “Swim” (unrelated action verbalization) evenly divided over 90 trials. The verbalizations were delivered 2 s before trial end, were equalized in volume level, and were recorded and configured to last exactly 0.5 s. The overall procedure is visualized in Figure 1.

### Temporal Binding

In the domains of temporal binding and agency, time perception is often measured either using the Wundt-clock paradigm (Haggard et al., 2002; Wohlschläger et al., 2003a,b; Engbert and Wohlschläger, 2007; Moore and Haggard, 2008; Moore et al., 2009a,b) or using the interval judgment task (Engbert et al., 2007, 2008; Cravo et al., 2009; Haering and Kiesel, 2014; Damen et al., 2015). In the Wundt-clock paradigm, conclusions about temporal binding are drawn based on the differences with the estimated and actual time events, using a virtual clock interface as an instrument to gauge time, and featuring baseline blocks to be able to correct for individual error. This allows not only for contrasts between experimental conditions but also for contrasts of subjective time compared to real-time. The interval judgment task in the present experiment does not feature comparable procedures to make the subjective experience more in line with objective reality. It is thereby well able to capture one’s subjective experience of time intervals, but it is also likely to show deviation from real-time (Buehner and Humphreys, 2009; Humphreys and Buehner, 2009). We therefore do not make comparisons between estimated and real-time intervals, and reported total interval estimates – not shifts from real-time. Temporal binding in the present study refers to relatively lower estimates in contrasts between experimental conditions, not to a reduction compared to the actual time intervals.

## Results

Means and SD’s of the experimental conditions in Studies 1–3 are reported in Table 1.

**Table 1:** Means and SD’s (in brackets) of the experimental conditions in Studies 1–3.

		Compatible cue	Opposite cue	Unrelated cue
**Study 1** (act)	2.5 s interval	2.736 (0.229)	2.693 (0.225)	
	3.0 s interval	3.023 (0.279)	2.996 (0.299)	
	3.5 s interval	3.418 (0.361)	3.349 (0.351)	
	Mean (SD)	3.059 (0.269)	3.013 (0.262)	
**Study 2** (observe)	2.5 s interval	2.852 (0.249)	2.821 (0.244)	
	3.0 s interval	3.106 (0.267)	3.099 (0.272)	
	3.5 s interval	3.399 (0.283)	3.414 (0.322)	
	Mean (SD)	3.119 (0.233)	3.111 (0.247)	
**Study 3** (act)	2.5 s interval	2.740 (0.334)	2.692 (0.341)	2.634 (0.328)
	3.0 s interval	3.031 (0.310)	2.986 (0.320)	2.913 (0.318)
	3.5 s interval	3.362 (0.344)	3.327 (0.330)	3.245 (0.358)
	Mean (SD)	3.066 (0.304)	2.909 (0.269)	2.990 (0.283)

*Numbers represent seconds. Actual time intervals were 3 s on average*.

### Study 1

A 3 (Time interval: 2.5 vs. 3.0 vs. 3.5 s) × 2 (Cue compatibility: compatible vs. opposite) repeated measures Analysis of Variance (ANOVA) on the average time estimates in Study 1 revealed a significant main effect of Cue compatibility, *F*(1, 49) = 8.507, *p* = 0.005, $\eta_{p}^{2}$ = 0.148. Participants’ time estimates were higher on the trials in which they heard a compatible cue, compared to the trials in which they heard an incompatible cue. As expected, there was also a main effect of the Time interval condition, *F*(2, 98) = 262.673, *p* < 0.001, $\eta_{p}^{2}$ = 0.843. Participants’ time estimates mapped the different trial intervals; estimates were smaller on the trials in which the timer interval was shorter, and higher on the trials in which the time interval was longer (*M*_2.5s_ = 2.715, SD = 0.212 vs. *M*_3.0s_ = 3.010, SD = 0.283 vs. *M*_3.5s_ = 3.384, SD = 0.346; all planned contrasts were statistically significant, *p*’s < 0.001; in all studies, linear approaches to the data were superior to quadratic approaches). There was no interaction effect between the Time interval and Cue compatibility conditions, *F*(2, 98) = 1.200, *p* = 0.306, $\eta_{P}^{2}$ = 0.024.

The compatibility effect was in line with our hypotheses and Study 1’s results provide support to the notion that overt and compatible action cues reduce agency compared to incompatible action cues (Damen et al., 2014).

### Study 2

Study 2 aimed to show that no compatibility effects emerge when individuals do not act. A 3 (Time interval: 2.5 vs. 3.0 vs. 3.5 s) × 2 (Cue compatibility: compatible vs. opposite) repeated measures ANOVA on the average time estimates in Study 2 indeed showed no main effect of Cue compatibility when individuals observed another agent and did not act themselves, *F*(1, 49) = 0.341, *p* = 0.562, $\eta_{P}^{2}$ = 0.007. Bayesian model averaging techniques using JASP (JASP Team, 2016; Wagenmakers et al., 2016) revealed a BFInclusion = 0.103, indicating that these data (strongly) support null-results over actual interaction effects. There was a main effect of Time interval, *F*(2, 98) = 200.352, *p* < 0.001, $\eta_{P}^{2}$ = 0.803. Shorter intervals led to lower time estimates compared to longer intervals (*M*_2.5s_ = 2.837, SD = 0.233 vs. *M*_3.0s_ = 3.102, SD = 0.262 vs. *M*_3.5s_ = 3.406, SD = 0.290; all planned contrasts were statistically significant, *p*’s < 0.001). There was no interaction between the Cue compatibility and Time interval conditions, *F*(2, 98) = 1.101, *p* = 0.337, $\eta_{P}^{2}$ = 0.022.

Although much caution must be taken when statistically contrasting effects from separate studies, we performed a 2 (Cue compatibility: compatible vs. incompatible within-subjects) × 2 (Action: acting vs. observing between-subjects) repeated measures ANOVA that included participants from both studies. This analysis showed a main effect of the Action condition, as participants who acted showed more binding compared to participants who only observed, (*M*_act_ = 3.036, SD = 0.255 vs. *M*_observe_ = 3.208, SD = 0.255; *F*(1, 98) = 11.173, *p* = 0.001, $\eta_{P}^{2}$ = 0.102). But more importantly, there was a significant interaction effect between the Cue compatibility and Action conditions, *F*(1, 98) = 107.395, *p* < 0.001, $\eta_{P}^{2}$ = 0.523. This interaction reflects the significant difference we observed of Cue compatibility when participants acted (S1), and the absence of a compatibility effect when participants did not act (S2).

### Study 3

Study 3 required participants to act and featured cues that were compatible, incompatible, or unrelated – thereby allowing us to investigate the direction of the compatibility effect. A 3 (Time interval: 2.5 vs. 3.0 vs. 3.5 s) × 3 (Cue compatibility: compatible vs. incompatible vs. unrelated) repeated measures ANOVA on the average time estimates in Study 2 revealed a significant main effect of Cue compatibility, *F*(2, 98) = 20.416, *p* < 0.001, $\eta_{P}^{2}$ = 0.294. Compatible cues led to higher time estimates than unrelated or opposite cues, and incompatible cues led to lower time estimates than unrelated and compatible cues (all contrasts were significant, *p’s* < 0.010). The analysis also revealed a significant main effect of Time interval, *F*(2, 98) = 146.964, *p* < 0.001, $\eta_{P}^{2}$ = 0.750. Shorter intervals led to lower time estimates compared to longer intervals (*M*_2.5s_ = 2.689, SD = 0.319 vs. *M*_3.0s_ = 2.977, SD = 0.297 vs. *M*_3.5s_ = 3.311, SD = 0.332; all planned contrasts were statistically significant*, p*’s < 0.001). No interaction effect between the Cue compatibility and Time interval conditions emerged, *F*(4, 196) = 0.197, *p* = 0.940, $\eta_{P}^{2}$ = 0.004.

## General Discussion

### Summary of Findings

The present line of studies showed that individuals’ time estimates were influenced by verbal cues. Even though these cues were contextual and participants were instructed not to be influenced by them, trials on which participants heard cues that were compatible with their actions were experienced as relatively longer, and trials on which participants heard cues that were in opposition or unrelated to their actions were experienced as relatively shorter. This effect only occurred when participants were acting (Studies 1 and 3), not when they passively observed the same actions and effects being caused by an external entity (Study 2).

### Theoretical Implications

A paper by Damen et al. (2014) showed that compatible supraliminal action primes presented before participants performed their actions led to lower explicit agency ratings than incompatible action primes. As binding in time perception has been linked to increases in the sense of agency, and separation in time perception has been linked to decreases in agency (Haggard et al., 2002; Engbert et al., 2007, 2008; Moore et al., 2009a,b), our results are in line with the results by Damen et al. (2014). Additionally, the present research expands on that study by showing similar effects can occur when actions are already ongoing and when the contextual action cues are presented during action performance.

The single-word-instructions in the present research arguably represent social cues in their most limited sense (Yoshie and Haggard, 2017). However, previous literature has shown that completely non-social cues can have very different effects on measures of binding and agency. For example, when actions are primed and cued by arrows and targets on the Flanker task, compatible primes have been shown to increase agency (or alternatively, incompatible primes decrease agency; Sidarus and Haggard, 2016). However, cues that signal the presence and/or wishes of other agents seem to influence agency very differently: they reduce agency when we perceive our actions to be in line with them (Damen et al., 2014; Caspar et al., 2016).

Temporal binding and agency emergence have both been explained through processes relating both premotor prediction (Haggard et al., 2002; Engbert and Wohlschläger, 2007; Waszak et al., 2012) as well as post-motor inferences (Wegner, 2002, 2003; Moore and Haggard, 2008). However, many studies investigating the relation between post-motor inferential mechanisms and binding featured manipulations prior to action performance (e.g., Desantis et al., 2011; Barlas and Obhi, 2013). Given that these premotor inferential manipulations may (also) influence premotor prediction (e.g., Rigoni et al., 2011), it was important to further validate the unique contribution of inferential mechanisms to temporal binding. We attempted to do this by using inferential cues that related to actions – not outcomes – by using manipulations that only occurred after action initiation, and, by presenting those cues in a context of ongoing action performance where people may rely more strongly on inferential cues in general (Wen et al., 2015). As such, we believe our results further support the notion that inferential mechanisms are indeed important to effects of temporal binding.

### The Use of Continuous Actions in Binding and Agency Research

Previous studies in the domains of binding and agency regularly required their participants to perform a single *short* action (e.g., a button-press), to observe a single event (e.g., a tone), and subsequently to report agency scores or measures of time perception. As far as we know, the present research is the first to link *implicit* agency and time perception in a task featuring an ongoing action. This approach was useful for our purposes, as we were able to show that action cues influenced time estimation. However, whether the mechanisms of temporal binding are actually similar for ongoing isometric action performance compared to discrete action performance needs to be addressed by future research.

### Limitations and Suggestions

Since participants always pressed the button there was a slight asymmetry to the conditions, as the “press” action cue condition was always compatible with participants’ actions. As such, it is possible that our results were caused by more than mere compatibility but were also driven by differences in the psychological processing of default versus non-default options. Investigating prime-action compatibility when presenting multiple action possibilities and comparing default and non-default options therefore seem intriguing and important avenues for future research to take.

### Conclusion

The perception of time may be one of the most pervasive yet subjective experiences in human consciousness (Benford, 1944; Angrilli et al., 1997; Eagleman, 2008). The present research adds to line of research on the subjective nature of our perception of time. It shows that our perception of time is influenced not only by our own actions but also by the compatibility of those actions with contextual cues.

## Data Availability Statement

Methodology and Data files are accessible via the Surfdrive repository at the following location: https://surfdrive.surf.nl/files/index.php/s/fEi1h0bbtnamWk2.

## Ethics Statement

The studies involving human participants were reviewed and approved by the Research Ethics Board of Utrecht University’s Faculty of Social Sciences. All participants provided their informed consent to participate in this study.

## Author Contributions

All authors listed have made a substantial, direct and intellectual contribution to the work, and approved it for publication.

### Conflict of Interest

The authors declare that the research was conducted in the absence of any commercial or financial relationships that could be construed as a potential conflict of interest.
